# Analysis of Incidental Gallbladder Cancer in Cholecystectomies

**DOI:** 10.7759/cureus.5710

**Published:** 2019-09-20

**Authors:** Murat Kanlioz, Ugur Ekici, Yaşar Ayva

**Affiliations:** 1 General Surgery, Beylikdüzü Kolan Hospital, Istanbul, TUR; 2 General Surgery, İstanbul Gelisim University, Istanbul, TUR; 3 Pathology, Sincan State Hospital, Ankara, TUR

**Keywords:** incidental gallbladder cancer, cholecystectomy, routine histopathological examination, gallbladder

## Abstract

Objective: To study incidental gallbladder cancer (IGBC) incidence in patients who underwent cholecystectomy.

Methods: The records of patients who underwent cholecystectomy between 2004-2019 were retrospectively reviewed. The demographic information, preoperative radiological findings of the patients diagnosed with gallbladder cancer (GBC), as a result of routine histopathological examination and operation records, were reviewed and findings were recorded. The preoperative radiological records of the patients with GBC and, if any, findings of GBC suspected during surgery were recorded.

Results: Between 2004-2019, a total of 6314 patients underwent cholecystectomy. Of the patients, 5404 (85.59%) were female and 910 (14.41%) were male. The median age was 47 years (min:19, max:94) and the mean age was 47.28±14.60 years. Nine out of 6314 patients (0.14%) were diagnosed with GBC by postoperative histopathological examination. All patients with GBC were female and their mean age was 64.33±11.08 years. Two out of nine GBC cases were prediagnosed with GBC in preoperative radiological findings; the remaining seven (0.11%) had IGBC without any preoperative findings.

Conclusion: Asian populations are reported to have a higher incidence of GBC. Turkey is located in the transition zone between Asia and Europe. However, the GBC rates in our study remain far below the rates reported in Asian publications. We believe that our results may be affected by the predominantly Mediterranean-type diet and the relatively higher socioeconomic level of the region where we conducted our study. Consequently, we recommend routine histopathological examination after cholecystectomies in regions with a high incidence of GBC.

## Introduction

Although not frequent, gallbladder cancers (GBC) rank fifth among gastrointestinal system cancers in terms of incidence. GBC has a lower rate of incidence, yet it is the most common cancers of bile ducts [1]. Adenocarcinoma is the most common type of histopathological types of GBCs. The ratio of adenocarcinoma to all other GBCs is about 90%. Adenocarcinoma mainly consists of the following three subtypes: papillary, tubular and mucinous. The histological types other than adenocarcinoma (anaplastic, squamous and adenosquamous carcinoma) have a quite low rate of incidence [2]. GBC appears to be 3-4 times more common in females compared to males. It is generally more common in patients over 60 years of age [3]. GBC is an aggressive tumor which has a five-year survival rate ranging from 5% to 20%. In most cases, the treatment outcomes are far from being satisfactory since it is diagnosed in late and advanced stages, hence resulting in high mortality and morbidity rates [4]. Whilst, in the preoperative period, GBC is diagnosed in only 30% of GBC cases, 70% of the GBC cases are diagnosed incidentally. Incidental gallbladder cancers (IGBC) are found during the routine histopathological examination mostly performed due to cholelithiasis (GBL), gallbladder polyps (GBP) and gallbladder infection (GBI) [4]. The rate of IGBC after cholecystectomies varies by geographic region and has been reported in different series with a rate between 0.25% and 3.3% [5-7].

## Materials and methods

If, for whatever reason, a surgery is performed in our hospital, all cholecystectomy materials are subjected to routine histopathological examination. The patients who underwent cholecystectomy between 2004 and 2019 were reviewed retrospectively. In our study conducted based on the patients' file information, we analyzed the cases which were diagnosed with IGBC following the routine histopathological examination of the gallbladders of the patients who underwent open or laparoscopic cholecystectomy due to GBL, GBI, and GBP without the suspicion of GBC in preoperative radiological examinations and check-up. Also, the file information of the patients who underwent surgery with the preliminary diagnosis of GBC were recorded. The demographic data of all patients who underwent cholecystectomy, the treatments administered, and their survival periods were analyzed. Besides, the data on the survival rates of the patients in the file were reviewed, and the patients with GBC and IGBC or their relatives were contacted to confirm the information obtained and to learn about any missing or suspicious cases. This study was conducted with the approval of the institute and informed consent from the patients.

## Results

The files of 6314 patients who underwent surgery for gallbladder diseases (GBD) between 2004 and 2019 were reviewed retrospectively. The study included the patients whose diagnosis and treatment data were completely available in the file. Of the 6314 patients who underwent cholecystectomy, 5404 (85.59%) were female and 910 (14.41%) were male. In patients who underwent cholecystectomy, the median age was 47 years (min:19, max:94) and the mean age, which was 47.28±14,60 years in all patients, was 47.60±12.35 years in females and 46.53±12.65 in males. A total of nine (0.14%) out of 6314 patients who underwent cholecystectomy were diagnosed with GBC and IGBC. The mean age of those nine patients was 64.33±11.08 years (min:41, max:79). Of a total of 6314 patients who underwent cholecystectomy, seven (0.11%), all of whom were female, were diagnosed with IGBC following the routine histopathological examination performed in the postoperative period. The mean age of those diagnosed with IGBC was 60.85±10.09 years (min:41, max:74). Two female patients, however, were taken into surgery with suspicion of GBC arose in the preoperative examination after tumor markers and radiological scans had been performed. The patients who were taken into surgery with suspicion of GBC were 74 and 79 years old. The postoperative histopathological examination results of two patients with suspected GBC were reported as GBC. The patients who were taken into surgery due to a preliminary diagnosis of GBC had gallbladder bed resection and regional lymphadenectomy combined with cholecystectomy. Also, the seven patients with IGBC underwent a reoperation on the 17th day on average and had gallbladder bed resection and lymphadenectomy. Of the nine patients with GBC, eight were reported to have adenocarcinoma and one was reported to have a malignant epithelial tumor. The patients with GBC and IGBC were consulted with the medical oncology department for adjuvant therapy, three of which subsequently underwent adjuvant chemotherapy. Of those patients, the mean length of hospital stay was 8.6 days. The mean survival period of the GBC and IGBC patients was found to be 19.2 months (2 months to 52 months). Of the patients with GBC, three are still alive (Table 1).

**Table 1:** Numbers, Rates, and Age Statistics of Patients GBC: gallbladder cancer; IGBC: incidental gallbladder cancer.

	Cholecystectomy	GBC	IGBC	GBC(+)IGBC
Total Number of Patients	6314	2(0.03%)	7(0.11%)	9(0.14%)
Mean Age (years)	47.28±14.60	76.5±2.5	60.85±10.09	64.33±11.08
Min. and Max. Age (years)	19 to 94	41 to 74	74 to 79	41 to 79
Number of Female Patients	5404(85.59%)	2(0.03%)	7(0.11%)	9(0.14%)
Number of Male Patients	910(14.41%)	0	0	0

## Discussion

We calculated the mean age of the patients diagnosed with GBC and IGBC as 64.33±11.08 years. Many publications report different mean ages for GBC. Besides, the mean age was reported to be 62.4 years in the study of Aldossary et al. and 52.4 years in the study conducted by Charfi et al. [8-10]. The mean age varies depending on regions and the number of cases in the study population. The literature shows that that male-to-female ratio in GBC patients is between 1/3 and 1/4. All GBC and IGBC cases that we identified in our study are female patients. The male-to-female ratio are reported to be 1/1.5 by Mochizuki et al., 1/3 by Wu et al. and 1/4 by Charfi et al. [9-11]. It is observed in studies with a high number of cases that the mean age and gender distribution is close to those stated in the literature, whereas it is striking that the male-to-female ratio in our study was 1/6 in all of our cholecystectomies and all our patients with GBC were females. This result suggests that further studies involving more patients or multi-center researches should be undertaken.

On the other hand, the rates of diagnosing GBC and IGBC in cases who underwent cholecystectomy appear to be significantly affected by regional differences. In our study, the numbers of GBC and IGBC were found to be two (0.03%) and seven (0.11%), respectively, whilst the total number was nine (0.14%). The number of IGBCs was reported to be seven (2.05%) in 341-case series by Tatli et al., 20 (0.41%) in 4800-case series by Jha et al., eight (2.3%) in 352-case series by Utsumi et al., and six (0.15%) in 4024-case cholecystectomy series by Patel et al. [6,12-14]. In the study conducted by Patel et al., the number of cases was 4024, being 6314 in our study, whereas the IGBC rates in both studies are close to each other. With reference to this, it is observed that the number of IGBCs is found to be low as the number of cases increases, and on the contrary, in studies involving a low number of cases, those values are found to be remarkably high. It is also remarkable that the rates reported by Tatli et al. in their study conducted in Turkey is about 15 times more than the rates that we identified. However, it may be suggested that the low number of cases in the study conducted by Tatli et al. (341) may have an impact on the high rate obtained. Also, we should state that the most important difference between the region where Tatli et al. conducted their study and the region where we conducted our study is the dietary habit. In the region where Tatli et al. conducted their study, the local people consume extremely fatty and spicy food as well as grilled meat products. It should also be noted that the socioeconomic level in the region where Tatli et al. conducted their study is relatively low. It is seen that IGBC rates are generally higher in Asian studies [13]. We believe that the higher rates of GBCs reported in the studies conducted in Asia are probably due to dietary habits and environmental conditions. The IGBC rate in our study is, however, below the literature averages. The incidence rate of IGBC in our country, Turkey, which is located between Asia and Europe was found to be quite low compared to those reported in Asian studies. We believe that, at this point, the dietary habits are important. The region where we conducted our study has a high socioeconomic level in our country, and also is dominated by the Mediterranean diet. On the other hand, all of our GBC patients underwent gallbladder-liver bed resection and lymphadenectomy. No mortality occurred during the operations. Three of our patients received postoperative chemotherapy as a complementary treatment. The mean length of hospital stay of our patients was 8.6 days. The mean survival period of our patients was 19.2 months (2 to 52 months). The literature reports various survival periods. Conducted by Ethun et al., a cohort study involving 10 centers reports that 207 (46%) of 449 patients with GBC had IGBC and their median survival period was 40.4 months [15]. In their study, Tian et al. reported that 69 (0.91%) out of their 7582 patients with GBL had IGBC, the mean age of IGBC patients was 61 years, and the survival rates were 89.9%, 78.3% and 76.8% in one-, three- and five-year follow-ups, respectively [16]. The survival rates reported by Ethun and Tian suggest that we should further improve ourselves in the treatment of GBC.

We see that there are serious debates on whether or not a histopathological examination should be performed on cholecystectomy materials following cholecystectomies. From the perspective of cost analysis, it is claimed that examination of the gallbladder specimens with no suspicion of cancer causes loss of money and time since the number of IGBC cases is low. Following their histopathological study, Shukla et al. recommended that a routine histopathological examination should be performed [17]. In their study, however, Mittal et al. reported that the routine histopathological examination of cholecystectomy materials would be unnecessary if there are no visually suspicious findings found in the preoperative imaging of all GBC cases or during the operation [18]. Moreover, Cavallaro et al. reported that the histopathological examination of cholecystectomy materials should not be performed routinely, but rather for only the selective cases [19].

## Conclusions

We believe that, due to the aggressive course of GBC, higher treatment costs in advanced stages, and the increase that occurs in survival rates in case of early diagnosis, cholecystectomy materials should be included in routine histopathological examination especially in geographies where GBC incidence is high.
